# Reciprocating Kinematics of X-Smart Plus, VDW Silver and, iRoot Endodontic Motors: A Comparison Between Real and Set Values

**DOI:** 10.1590/0103-6440202204855

**Published:** 2022-12-05

**Authors:** Dieimes Braambati, Renata de Castro Monteiro, Marcelo Santos Coelho, Adriana de Jesus Soares, Marcos Frozoni

**Affiliations:** 1 Endodontic Department, São Leopoldo Mandic School of Dentistry, Campinas- SP, Brazil; 2 Endodontic Department, State University of Campinas, Piracicaba- SP, Brazil

**Keywords:** Reciprocating, endodontic motor, kinematics, video analysis

## Abstract

This study assessed 3 endodontic motors, X-Smart Plus (Dentsply Sirona, Ballaigues, Switzerland), VDW.Silver Reciproc (VDW GmbH, München, Germany) and, iRoot (Bassi Endodontics, Belo Horizonte, Brazil) in 2 different reciprocating settings. The movements evaluated were 170° in counter-clockwise (CCW) and 50° in clockwise (CW) at 350 RPM, and 150° CCW and 30° CW at 300 RPM. For the X-Smart Plus and VDW Silver the settings used were the ones in the motor library. For the iRoot, the motor was adjusted to the angles of the study. A customized optic target was attached to the contra-angle of the motor and the movements were recorded with a high-resolution camera (K2 DistaMaxTM Long-Distance Microscope System, Infinity Photo-Optical Company, Colorado, EUA) at 2,400 frames per second (FPS). The images were analyzed with the Vision Research software (Inc. Headquarters, Wayne, New Jersey, EUA). The following kinematic parameters were assessed: CCW angle, CW angle, speed (RPM) at both directions, and, standstill time at each change of directions. The Intraclass Correlation Coefficient (ICC) and Kruskal-Wallis (method of Dunn) were used at a significant level of 5%. There was no statistically significant difference among the motors at the 150°/30° setting (P > .05); the iRoot was the least reliable at the 170°/50° setting for CCW angle, speed, and net angle parameters (P < 0.05). The standstill time of all motors in both directions was identical. None of the motors were able to reproduce faithfully the set movements. The iRoot motor presented a higher discrepancy when compared to X-Smart and VDW Silver.

## Resumo

Este estudo avaliou 3 motores endodônticos, X-Smart Plus (Dentsply Sirona, Ballaigues, Suíça), VDW.Silver Reciproc (VDW GmbH, Munique, Alemanha) e iRoot (Bassi Endodontics, Belo Horizonte, Brasil) em 2 diferentes ajustes de movimento reciprocante. Os movimentos analisados foram: 170° em sentido anti-horário (CCW) e 50° em sentido horário (CW) a 350 RPM, e 150° CCW e 30° CW a 300 RPM. Para os motores X-Smart Plus e VDW Silver os ajustes usados foram os que se apresentam nos ajustes pré-definidos dos motores. Para o iRoot o motor foi ajustado para os ângulos do estudo. Um alvo ótico customizado foi preso ao contra ângulo do motor e os movimentos foram gravados com uma câmara de alto resolução (K2 DistaMaxTM Long-Distance Microscope System, Infinity Photo-Optical Company, Colorado, EUA) a 2.400 quadros por segundo (FPS). As imagens foram analisadas com o sotware Vision Research (Inc. Headquarters, Wayne, Nova Jersey, USA). Os seguintes parâmetros de cinemática foram avaliados: ângulo anti-horário, ângulo horário, ângulo líquido, velocidade (RPM) em ambas direções e tempo de parada a cada mudança de direção. O Coeficiente de Correlação Intraclasse (ICC) e o teste de Kruskal-Wallis (método de Dunn) foram usados com nível de significâncias de 5%. Não houve diferença estatisticamente significante entre os motores no ajuste de 150°/30° (P > .05); o motor iRoot foi o menos confiável no ajuste de 170°/50° para o ângulo anti-horário, velocidade e ângulo líquido (P < 0.05). O tempo de parada em todos os motores foi idêntico em ambas as direções. Nenhum dos motores foi capaz de reproduzir fielmente os movimentos. O motor iRoot apresentou maior discrepância quando comparado com o X-Smart Plus e VDW Silver.

## Introduction

The removal of pulp tissue and/or biofilm is one of the main steps in root canal therapy. This complex task is achieved with irrigation and metallic endodontic instruments scraping the root canal walls ^1^. Several different instruments have been proposed for root canal shaping, especially after the introduction of Nickel-titanium alloy in endodontics ^2^. In addition to the different systems, there are different kinematics that can be used in these instruments ^3^. The reciprocating kinematics was introduced in endodontics aiming for a safer and faster root canal preparation while opening the possibility of single-file preparation ^4^ ^,^ ^5^. The initial proposal, used a file originally designed for rotary motion, the ProTaper F2. This instrument was used in a 144° clockwise (CW) movement with a counter-clockwise (CCW) of 72° ^4^. This kinematics has been extensively studied in recent years showing an increase in cyclic fatigue resistance of the files and centered preparation ^6^ ^-^ ^8^.

Thereafter, some reciprocating root canal preparation systems were launched with some changes. Instead of a conventional rotary file, a dedicated reciprocating file was indicated, now, using reverse blades making its action in the CCW direction ^9^. The engaging movement was at a CCW angle and, the disengaging action was at a CW movement. The most common set of movements is 170°/50° at 350 RPM for WaveOne (Dentsply Sirona, Ballaigues Switzerland) and, 150°/30° at 300 RPM, VDW (VDW, Munich, Germany) ^10^. These movements resulted in a net angle of the movement of 120°, meaning that every cycle of 3 movements culminates in a full rotation of the file.

The reliability of these angles is important to the clinician due to some impacts on the instrument behavior and root canal preparation. Shorter angles of reciprocation produce increased cyclic fatigue resistance of the instrument, more centered preparation, and less canal transportation ^11^. On the other hand, the preparation time increases at a shorter reciprocation angle ^11^ ^,^ ^12^. A recent study reported that a short angle of reciprocation could eliminate the risk of separation of the instrument (stainless steel or NiTi) for glide path creation in reciprocating movement ^13^.

Aiming to increase the possibilities of instrumentation usage, some recently launched endodontic motors present the possibility of adjustment of the angles by the clinician. It is possible to use instruments created for rotary kinematics in a reciprocation motion by altering the reciprocating angles. This personalization opens the possibility for the clinician to adjust the motor depending on the proposal of a specific case. However, a previous study has already questioned the reliability of some endodontic motors in regards to the actual angle of reciprocating and the rotational speed ^14^. These new adjustable endodontic motors are even less known in regards to the actual angles of reciprocation and speed delivered.

Therefore, this study aimed to assess the actual parameters of 3 different endodontic motors set at 2 kinematics proposed for reciprocating endodontic systems. Two endodontic motors, already available for several years, with set angles and speed, X-Smart Plus (Dentsply Sirona) and, VDW Silver (VDW), and a recently launched adjustable motor iRoot (Bassi Endodontics, Belo Horizonte, Brazil) were tested. The null hypothesis tested is that there is no difference among the tested motors.

## Materials and Methods

The sample size calculation was done based on the results of the X-Smart Plus tested by Irmak & Orhan ^15^. To achieve an Intraclass Correlation Coefficient (ICC) of at least 0.75, a significance level of 5%, and a power of 80%. The calculation required that each recording of 10 seconds was repeated 3 times in each sample. The spreadsheet Excel 2013 (Microsoft, Seattle, WA) was used for the calculation.

### Endodontic motor selection and setting

The 3 endodontic motors used were the X-Smart Plus (Dentsply Sirona, Ballaigues, Switzerland), VDW Silver (VDW, Munich, Germany), and, iRoot (Bassi Endodontics, Belo Horizonte, Brazil), The movement settings testes were 150° CCW and 30° CW at 300 RPM and, 170° CCW and 30° CW at 350 RPM, resulting in 6 different groups as follows:

Group X-Smart/WaveOne mode - X-Smart motor 170°/50° at 350 RPM
Group X-Smart/Reciproc mode - X-Smart motor 150°/30° at 300 RPM
Group VDW - WaveOne mode - VDW motor 170°/50° at 350 RPM
Group VDW/Reciproc mode - VDW motor 150°/30° at 300 RPM
Group iRoot/WaveOne adjustment - iRoot motor 170°/50° at 350 RPM
Group iRoot/Reciproc adjustment - iRoot motor 150°/30° at 300 RPM

The X-Smart and VDW motors were used at the Reciproc Reciprocating ALL mode or WaveOne Reciprocating ALL mode available in the library of the motors accordingly to the group of choice. Meanwhile, the iRoot motor was manually adjusted to the angles and speed for each group. All motors operated with the battery fully charged and unplugged from the power cord. In addition, it was checked that the motors were calibrated before each use.

### Kinematics analysis

**Figure 1:**
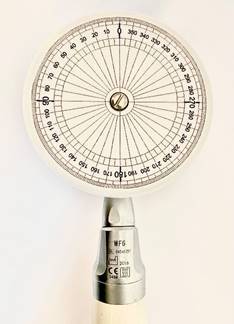
The 5-cm Polypropylene disk with registration from 0° - 360° with intervals at each degree used in the experiment

The assessment of each group’s real angles and speed was made with the aid of a customized polypropylene target with 5 cm of diameter with 359 lines marked representing each degree, based on a previous study ^14^. This target was attached to a disk and then attached to the contra-angles of each motor (Figure 1).

**Figure 2.**
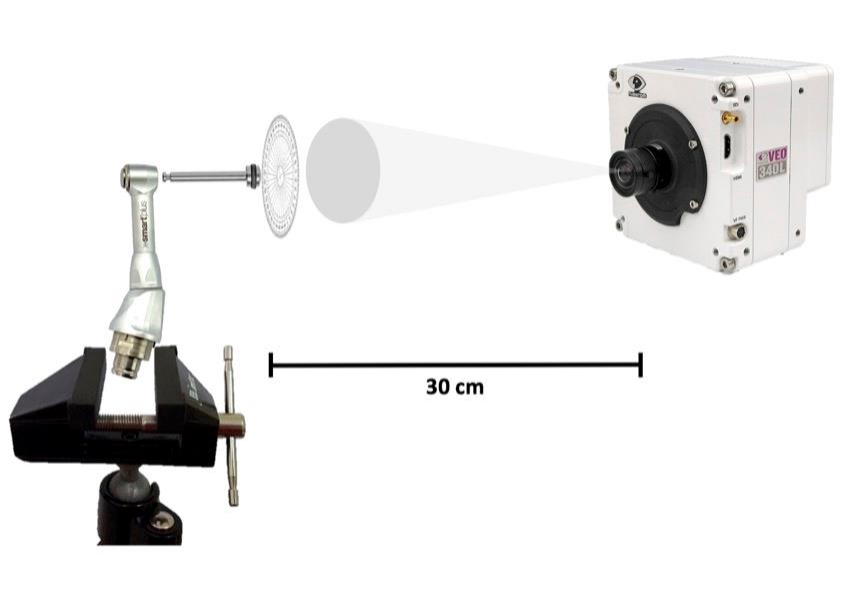
Set of the camera, lens, target, contra-angle, and water-aligned device used for the registration of the images

The images were obtained with a super macro lens, model M317111, with a focal adjust of 0.5 coupled to a high-speed camera (Phantom VEO 710, Infinity Photo-Optical Company, NJ). The lens was placed at 30 cm from the target and the device was aligned with a water device (Figure 2). The camera was adjusted for high-resolution video at 2,400 frames per second (FPS) and a resolution of 800 x 800 pixels. Each group was recorded in the CINE format, in 3 repetitions of 10s under illumination of 7,000 lumens (GS Vitec SN: 6500068, GS Vitec GmbH, Bad Soden-Salmünster, Germany). Then, 10 cycles of sequential reciprocating movements were randomly selected and analyzed using the software (Vision Research ®, Inc Headquarters, Wayne, NJ). The following data were obtained:

1. Duration of the CCW movement (milliseconds): Dccw;
2. Duration of standstill after CCW movement (milliseconds): Sccw;
3. Duration of the CW movement (milliseconds): Dcw
4. Duration of standstill after CW movement (milliseconds): Scw
5. Engagement (CCW) angle (°): ∝e;
6. Disengagement (CW) angle (°): ∝d.

After the collection of these data the following parameters were calculated:

1. Net angle of the cycle (°):
∝e-∝d;
2. Engagement speed (RPM):
$$\;\frac{\propto e}{Dccw}\;x\frac{60000ms}{360{}^{\circ}}$$;
3. Disengagement speed (RPM):
$$\;\frac{\propto d}{Dcw}\;x\frac{60000ms}{360{}^{\circ}}$$;
4. Speed of the total reciprocating cycle (RPM):
$$\frac{\propto e+\propto d}{Dccw+Sccw+Dcw+Scw}\;x\frac{60000ms}{360{}^{\circ}}$$;
5. Number of cycles to complete a full rotation:
$$\;\frac{360{}^{\circ}}{net\;angle}$$

### Statistical analysis

The Intraclass Correlation Coefficient was used to assess the replicability of the measurements done by the evaluator and compare the set and the actual values for each motor. For the comparison among the 3 motors and each set, the Kruskal-Wallis (post-hoc Dunn) were used. The SPSS 23 (SPSS, INC., Chicago, IL) program was used at a significance level of 5%.

## Results

The reliability of the evaluator showed excellent results for all parameters at all intervals, except for the Scw - duration of standstill after returning rotation that showed moderate reliability on day 1 (Table 1).

**Table 1:** Intraclass Correlation Coefficient for the different parameters, the asterisk indicates the results with moderate reliability, all the other results presented excellent reliability

Comparison	EA	Sccw	DA	Dcw	Dccw	Scw
Day 1 x Day 2	1.000	0.816	0.999	0.999	1.000	0.671*
Day 1 x Day 3	1.000	0.816	0.999	0.999	1.000	0.671*
Day 1 x Day 4	1.000	0.816	0.999	0.999	1.000	0.671*
Day 1 x Day 5	1.000	0.816	0.999	0.999	1.000	0.671*
Day 2 x Day 3	1.000	1.000	1.000	1.000	1.000	1.000
Day 2 x Day 4	1.000	1.000	0.999	1.000	1.000	1.000
Day 2 x Day 5	1.000	1.000	1.000	1.000	1.000	1.000
Day 3 x Day 4	1.000	1.000	0.999	1.000	1.000	1.000
Day 3 x Day 5	1.000	1.000	1.000	1.000	1.000	1.000
Day 4 x Day 5	1.000	1.000	0.999	1.000	1.000	1.000

*EA - engagement angle (CCW); Sccw - duration of standstill after advance; DA - disengagement angle (CW); Dcw - duration of returning rotation; Dccw - Duration of advancing rotation; Scw - duration of standstill after returning rotation*

The results of ICC showed that all motors had poor reliability when the real values were compared to the set values. At the WaveOne adjustment, the iRoot presented an engagement angle significantly higher than X-Smart Plus and VDW Silver (p = 0.027); the disengagement angle was similar among the motors (p = 0.067). At the Reciproc adjustment, the motors presented no statistically significant difference for both engagement (p = 0.063) and disengagement angles (p = 0.063). Table 2 depicts the actual values, errors, and reliability of each motor.

At the WaveOne adjustment, when compared to X-Smart Plus and Reciproc, the iRoot motor presented statistically significant different values for time required to engage (p=0.027), net angle of movement (p= 0.027), and consequently lower number of cycles to complete a full rotation (p = 0.021).

**Table 2:** Results for each motor, mean (standard deviation) in regards to set engagement (CCW) and disengagement angle (CW) and actual values and differences. CCI test showed poor reliability values for all motors.

	Set angle		Actual angle		Absolute errors and percentages in comparison with the set values		Reliability
	CCW	CW	CCW	CW	CCW	CW	
X-Smart Plus WaveOne mode	170°	50º	156.8 (0.4) A	56.8 (0.3) A	-13.2º (0.4º) -7.7% (0.2%)	6.8º (0.3º) 13.7% (0.6%)	CCW: CCI=0.000 CW: CCI=0.186
X-Smart Plus Reciproc mode	150°	30º	161.1 (1.3) a	53.9 (0.4) a	11.1º (1.3º) 7.4% (0.8%)	23.9º (0.4º) 79.7% (1.2%)	
VDW.Silver WaveOne mode	170°	50º	158.8 (0.3) A	56.8 (0.4) A	-11.2º (0.3º) -6.6% (0.2%)	6.8º (0.4º) 13.6% (0.9%)	CCW: CCI=0.000 CW: CCI=0.173
VDW.Silver Reciproc mode	150°	30º	160.5 (1.7) a	54.1 (0.2) a	10.5º (1.7º) 7.0% (1.1%)	24.1º (0.2º) 80.2% (0.5%)	
iRoot - WaveOne Adjustment	170°	50º	285.6 (17.6) B	130.8 (2.1) A	115.6º (17.6º) 68.0% (10.4%)	80.8º (2.1º) 161.6% (4.1%)	CCW:CCI=0.094 CW:CCI= 0.140
iRoot- Reciproc Adjustment	150°	30	238.6 (17.3) a	94.5 (2.7) a	88.6º (17.3º) 59.1% (11.6%)	64.5º (2.7º) 215.1% (8.9%)	

Different uppercase letters indicate statistically significant differences among motors for the 170°/50° movement (p < 0.05). Different lowercase letters indicate statistically significant differences among the motors for the 150°/30° movement at p < 0.05.

At the Reciproc adjustment, the iRoot motor was significantly different from X-Smart and VDW only at the duration of the disengage rotation (p = 0.027). For all other parameters, the motors were similar among them (Table 3).

**Table 3:** Results, mean (standard deviation) for each motor in all calculated parameters.

Parameter	X-Smart Plus		VDW.Silver		iRoot	
	WaveOne mode	Reciproc mode	WaveOne mode	Reciproc mode	WaveOne Adjustment	Reciproc Adjustment
Duration of the CCW movement (milliseconds)	74.28 (0.23) A	86.39 (0.52) ^a^	75.25 (0.34) A	85.76 (0.82) ^a^	124.03 (2.16) B	122.84 (2.62) ^a^
Duration of standstill after CCW	0.83 (0.00) A	0.83 (0.00) ^a^	0.83 (0.00) A	0.83 (0.00) ^a^	0.83 (0.00) A	0.83 (0.00) ^a^
Duration of the CW movement	32.53 (0.10) A	34.62 (0.12) ^a^	32.46 (0.18) A	34.83 (0.04) ^a^	94.23 (35.21) A	54.32 (3.02) ^a^
Duration of standstill after CW	0.83 (0.00) A	0.83 (0.00) ^a^	0.83 (0.00) A	0.83 (0.00) ^a^	0.83 (0.00) A	0.83 (0.00) ^a^
Net angle of movement	100.00 (0.10) A	107.23 (1.33) ^a^	102.00 (0.10) A	106.40 (1.57) ^a^	154.80 (15.58) B	144.07 (14.67) ^a^
Speed CCW	351.91 (0.32) A	310.87 (0.89) ^a^	351.72 (1.61) A	311.84 (0.99) ^a^	384.14 (30.23) A	324.13 (30.34) ^a^
CW speed	291.21 (1.92) A	259.45 (0.86) ^a^	291.66 (0.75) A	258.70 (0.69) ^a^	251.31 (80.17) A	290.36 (8.10) ^a^
Cycle speed	328.30 (0.43) A	292.14 (0.75^) a^	328.54 (1.22) A	292.45 (0.73) ^a^	322.64 (62.89) A	310.45 (17.81) ^a^
Cycles to complete full rotation	3.60 (0.00) B	3.36 (0.04) ^a^	3.53 (0.00) B	3.38 (0.05) ^a^	2.34 (0.24) A	2.52 (0.25) ^a^

Values with different uppercase letters indicate statistically significant differences for the 170°/50° movement (WaveOne) (p < 0.05). Values with different lowercase indicate statistically significant differences for the 150°/30° movement (Reciproc) at p < 0.05.

## Discussion

The mechanical automated preparation of root canals is most frequently achieved with NiTi rotary instruments. These instruments can be used in both rotary or reciprocating kinematics. However, the latter kinematics seems to be more complicated to be reliably reproduced. Also, the assessment of this kinematics requires a more detailed methodology. Fidler ^14^ used a high-speed camera with 1,000 FPS and 224 x 64 pixels and Irmak & Orhan used a camera with 1,200 FPS and 336 x 96 pixels ^15^. Aiming to achieve the best precision possible, the present study used a camera with 2,400 FPS and a resolution of 800 x 800 pixels, which can be considered significantly better than the aforementioned studies.

The poor reliability of the present study in regards to the reciprocating angles of X-smart Plus and VDW Silver is in agreement with previous studies ^14^ ^,^ ^15^. However, these studies were focused on endodontic motors with pre-set angles and speed. To the best of our knowledge, the present study is the first to assess the reliability of the iRoot motor, which permits the adjustment of the angles. Our findings suggest that, in agreement with the findings of previous studies with different motors, this motor is not reliable in regards to the angles of reciprocation when set at the WaveOne mode. Moreover, the results of iRoot were more discrepant than X-Smart and VDW Silver, thus, the null hypothesis was rejected.

In this study, the VDW Silver presented actual CCW and CW similar to the ones presented by Fidler ^14^: 159.85° ± 1.04 / 41.44 ± 1.49 at 343.36 ± 2.81 RPM at the WaveOne set and 158.6 ± 1.56 / 34.65° 1.13° at 282.92 ± 3.70 RPM the Reciproc mode. Likewise, the X-Smart Plus in the present study presented results corroborating Irmad and Orhan ^15^ - 151,70° ± 3,48º/ 62,00° ± 2,45º, 351,7 ± 7,07 RPM in the WaveOne mode and, 153,90° ± 8,57º / 58,85° ± 2,90º at 301,5 ± 6,73 RPM at the Reciproc mode. One of the possible reasons for this slight discrepancy in the results might be the differences in FPS and the number of pixels in the images among the studies.

It was demonstrated that a difference in the angles at the order of 30° might compromise the center ability of preparations ^11^. Despite the poor reliability, X-Smart Plus and VDW Silver were similar in their accuracy and none of these motors presented a discrepancy of the set angles higher than 30°. The greatest imprecision was related to the disengagement angle which was as high as 24.1° (80.2%) for the VDW Silver at the Reciproc mode. Also, the X-Smart Plus presented a 23.9° (79.7%) of discrepancy for the disengagement angle at the Reciproc mode, which seems to be acceptable. On the other hand, the iRoot motor presented values at least 64.5° (215.1%) discrepant from the set ones for the disengagement angle at Reciproc adjustment. This discrepancy reached 115.6° in the engagement angle at WaveOne adjustment. The errors in these angles resulted in a net angle of the cycle different from the 120° of the set movement. However, the iRoot motor at the WaveOne adjustment was less precise than the other motors, which can affect the centering ability of the preparations at this adjustment. The consequences of the CCW and CW angles are net angles and the number of cycles to complete a full rotation. At the set angles, it was expected that a full rotation should be completed at every sequence of 3 movements. Again, none of the motors was able to achieve what was adjusted; however, the iRoot motor at the WaveOne mode was the least reliable for the number of cycles to complete a full rotation.

A previous study demonstrated that increasing the CW angle reduced the cyclic fatigue resistance of instruments, theoretically, increasing the risk of instrument separation ^12^. Also, Santos et al ^16^ showed that reciprocating glide path instruments might separate at different angles. Therefore, submitting an instrument to a kinematic angle higher than the plastic deformation limit of the instrument increases the risk of fracture. The iRoot motor presented CW angle of 161.6% larger at the WaveOne adjustment and 215.1% larger at the Reciproc adjustment. The other motors presented at most 80.2% of discrepancy at the CW angle. Therefore, it can be concluded that the iRoot motor is riskier than the other motors when the instrument separation is considered.

A recent study showed the impact of the power of the rotating motors on the accuracy of the kinematics ^17^. Both X-Smart and VDW present a case with a battery and a cable connecting to the handpiece. Meanwhile, the iRoot is a wireless device, which potentially improves ergonomics. Also, both X-Smart and VDW motors present preset angles, while the iRoot it is possible to adjust these angles at its discretion. Both characteristics present a rationale; however, the failure to deliver adequate angles might compromise important features of the motor. The ability of the batteries in keeping the motors operating accurately should be the object of further studies.

At every change in the direction from CCW to CW and vice versa, there is a small time in which the motor standstills. Assessing the standstill time of the VDW Silver motor, Fidler reported values ranging from 3.1 ± 0.3 ms at the WaveOne mode up to 6.4 ± 0.8 at the Reciproc mode, meanwhile, other studies showed for the X-Smart Plus standstill times ranging from 1.21 ± 0.51 ms to 2.38 ± 1.65 ms. This fraction of seconds is due to the standstill necessary to change direction and might be affected by loose mechanical parts. Interestingly, in the present study, the time required for this change was the same,0.83 ms, for all 3 motors in all changes of directions. The fact that only unused motors were used in the present study could have an impact on the results.

In regards to the speed of engagement and disengagement, the results seem to be satisfactory. All motors presented similar values in both directions. Which is similar to previous findings ^14^ ^,^ ^18^. It seems that while the adjustment of the angles is critical, the speed delivered by the motors is not ideal, but slightly better than the angles.

The reciprocating movement opens a great variability of adjustments that can be used for the clinician either to improve safety, centrality, or speed of the preparation. However, it seems that no endodontic motor currently available can faithfully reproduce what the clinician is aiming for. One limitation of the present study is that all sets of movements were obtained without torque. This same limitation is found in the methodology used in previous studies; therefore, it might not reflect the challenges the motors are subject to in a clinical setting ^15^ ^,^ ^19^. In the present study, only 1 motor of each manufacturer was tested. Even though only brand-new motors were used, the specific motors tested might not reflect the overall findings from the total motors commercially available. Therefore, future studies including more samples of the motors are necessary.

## Conclusion

The present study showed that the actual values of the tested motors differ considerably from the set values. The iRoot endodontic motor presented a higher discrepancy when compared to the X-Smart Plus and VDW endodontic motors.
